# Control of *Erwinia amylovora* Growth by *Moringa oleifera* Leaf Extracts: In Vitro and in Planta Effects

**DOI:** 10.3390/plants11070957

**Published:** 2022-03-31

**Authors:** Riccardo Fontana, Giovanna Macchi, Anna Caproni, Mariaconcetta Sicurella, Mattia Buratto, Francesca Salvatori, Mariangela Pappadà, Stefano Manfredini, Anna Baldisserotto, Peggy Marconi

**Affiliations:** 1 Department of Chemical, Pharmaceutical and Agricultural Sciences, University of Ferrara, 44121 Ferrara, Italy; riccardo.fontana@unife.it (R.F.); anna.caproni@unife.it (A.C.); mattia.buratto@unife.it (M.B.); francesca.salvatori@unife.it (F.S.); mariangela.pappada@unife.it (M.P.); 2 Department of Life Sciences and Biotechnology, University of Ferrara, 44121 Ferrara, Italy; giovanna.macchi@edu.unife.it (G.M.); anna.baldisserotto@unife.it (A.B.); 3 Department of Environmental Sciences and Prevention, University of Ferrara, 44121 Ferrara, Italy; mariaconcetta.sicurella@unife.it

**Keywords:** *Erwinia amylovora*, *Moringa oleifera* Lam, plant extracts, antimicrobial activity, amylovoran, biofilm, crop protection, phytopathogen control

## Abstract

*Erwinia amylovora* (EA) is a phytopathogenic bacterium, the causative agent of bacterial fire blight, a disease that affects Rosaceaes. In order to replace antibiotics and copper, the antimicrobial activity of three extracts of *Moringa oleifera* Lam., methanolic (MeOH-MOE), hydroalcoholic (HA-MOE) and hydroalcoholic with maltodextrins (HAMD-MOE), was tested on eleven strains of EA isolated from apple trees by the Emilia-Romagna Phytosanitary Department. MIC and MBC have been evaluated; biofilm formation, swarming motility and amylovoran production were performed with the crystalviolet, soft-agar assay and the amylovoran method. All extracts demonstrated bacteriostatic activity at a concentration of 1 mg/mL, resulting in a 80% reduction in biofilm formation. HAMD-MOE, MeOH-MOE and HA-MOE caused an inhibition of motility of 60%, 65% and 30% after 6 days and a decrease in amylovoran synthesis of 84%, 63% and 93%, respectively. In planta results showed how the compounds were able to inhibit EA virulence on apple trees, mainly if they were applied as a preventive treatment, although the treatment showed a significant reduction in fire blight symptoms progression. The antibacterial activity of the extracts is mainly due to the high concentration of polyphenolic compounds detected in the extracts that was able to alter the permeability of bacterial membrane, resulting in slowing the synthesis of ATP and consequently of all ATP-dependent functions, such as motility and less selectivity towards harmful compounds, which can, thus, enter the cytoplasm and inhibit enzymes involved in replication and quorum sensing. The efficacy, eco-compatibility and low cost make such extracts a potential tool for the control of bacterial fire blight.

## 1. Introduction

*Erwinia amylovora* (EA), the causative pathogen of fire blight, is a phytobacteria of major economic importance in most *Rosaceaes* growing regions of the world. EA is a gram-negative enterobacterium, and it overwinters in living tissues at the margins of cankers and becomes active in spring when suitable climatic conditions are reached. The bacterium is transmitted to healthy tissues mainly by insects, wind and rain. The optimum climatic conditions for the multiplication of the bacterium are between 23 and 30 °C. The bacterium firstly infects the flowers of the host, thanks to the type III secretion system, primarily during the blooming period of trees. At this stage, symptoms are the typical “shepherd’s crook” of the twigs and a yellowish bacterial exudate on the infected tissues, called ooze (a mass of EA cells within an exopolysaccharide matrix) [1,2]. These oozes have been examined and the population size of EA has been determined to be 10^8^ CFU/µL of ooze [3]. The inoculum is followed by an epiphytic growth that helps EA to establish a large population, needed for infection, followed by a systemic spread (endophytic growth) through the plant xylem. The production of ooze may be the source for a second inoculum and for the spread of the disease, enhanced by the formation of plant cankers to tolerate winter temperatures.

To efficiently infect plants, EA needs virulence factors, and many have been characterized: to effectively establish infection, EA uses a complicated regulatory network in order to detect relevant environmental signals and, thus, coordinate the expression of early and late-stage virulence factors (including quorum sensing). The main factors described are the Type 3 Secretion System (T3SS), the exopolysaccharide (EPS) amylovoran, biofilm formation and motility [4]. Among these, amylovoran, an heteropolymer composed of a branched repeating unit consisting of galactose, glucose and pyruvate residues, plays an essential role, as it is fundamental for biofilm formation and endophytic growth [1]. Amylovoran is a pathogenicity factor because amylovoran-deficient mutants are avirulent, and, in addition, the quantity of amylovoran produced by individual EA strains is correlated with the degree of virulence, with weak producers exhibiting reduced virulence [5].

One of the main problematic aspects of fire blight is the lack of efficient control measures to stop its dissemination [6]. Current methods rely on preventive measures, such as fertilization, irrigation, shortening of the blooming period and pruning of the infected trees [7]. The control of EA by the use of copper-based compounds has resulted in phytotoxicity associated with high doses of copper in the soil and the acquisition of resistance by bacteria [8]. Streptomycin has been used to control fire blight, but the lack of efficacy at lower doses, high production cost and, mainly, the associated emerging antibiotic resistance, led many regions (e.g., European Union) to ban their use, although they are still allowed in North America and other countries [9]. The limitations in the control of this pest and the need to reduce antibiotics use in agriculture highlighted the urge for new, safe, and efficient control strategies. In response to this, one of the most studied fields in the last years is the identification of bacteriophages and antagonistic bacteria [10,11], and the application of essential oils and natural antimicrobial compounds [12].

Several investigations suggested that *Moringa oleifera* Lam. acts as an antibacterial agent on Gram-negative and Gram-positive bacteria [13,14,15]. *M. oleifera* Lam. phytocomplex is mainly composed of flavonoids, phenolic acids, alkaloids, isothiocyanates, tannins and saponins, which are responsible for its antimicrobial activity [16]. All the compounds found in *M. oleifera* Lam. extracts (MOEs) participate in the countless properties that the plant can offer. Flavonols such as quercetin, kaempferol and rutin and flavones, such as apigenin, are observed in high concentrations [17,18], as well as several glycosylated flavonoids, including quercetin-3-O-glucoside, kaempferol-3-O-glucoside and kaempferol-3-O-rutinoside [19]. Leaves are rich in phenolic acids such as gallic acid, chlorogenic acid, ellagic acid, ferulic acid and caffeic acid [20]. Numerous alkaloids were found in *M. oleifera* Lam. leaves, including marumoside A, marumoside B, α-L-ramnopiraonsyl-vincosamide, phenylacetonitrile and its glucopyranosilic derivative [21]. The dried leaves are an excellent source of carotenoids and retinol; and precursors of vitamin A, vitamins B, vitamin E and vitamin C, conferring antioxidant properties to *M. oleifera* Lam. [22].

In terms of antimicrobial activity, a number of studies have highlighted this property for different MOEs; i.e., in 2016, it was demonstrated how different extracts from seeds, leaves and roots were able to inhibit *Staphylococcus aureus* and *Streptococcus mutans* growth [23]; as for phytopathogens, our previous study shows how these extracts can inhibit the growth and virulence of different *Xanthomonas campestris* pv. *campestris* strains [24].

Given the economic impact that the disease causes in many regions, combined with the research of alternative treatments relative to the use of chemicals or antibiotics, we have an ongoing research program (SUSTANIA) aimed to explore the effects of different plant extracts, selected on the basis of their sustainable source and safe design (renewable plant parts, not at risk of extinction, high productivity and possibly used for food or cosmetics). In this context, from our previous research [24,25], we decided to study the effect of various extracts of leaves of *M. oleifera* Lam. on the virulence factors of EA: in particular, we focused on its in vitro effect on membrane permeability, swarming motility, amylovoran production and biofilm formation; and on in planta preventive and therapeutic effects.

## 2. Results

### 2.1. Determination of MICs of MOEs

The determination of the MIC of MOEs against EA was tested by the microplate assay. From our analysis, water extracts, such as the infusion (In-MOE) and the aqueous extract with maltodextrins (WMD-MOE), showed no efficacy at the tested concentrations (Table 1). As shown in Table 1, it was clear that both In-MOE and WMD-MOE did not express antimicrobial activity as they were neither bacteriostatic nor bactericidal at the highest concentration tested. On the other hand, HA-MOE, MeOH-MOE and HAMD-MOE showed complete inhibition of bacterial growth at 1 mg/mL. The greatest activity of the alcoholic extracts is closely related to the manner they are extracted, as the use of solvents such as ethanol or methanol allows, according to the principle of molecular affinity, obtaining more concentrated extracts in terms of bioactive molecules constituting the phytocomplex.

### 2.2. In Vitro Assessment of Membrane Permeability Alteration

**Figure 1:**
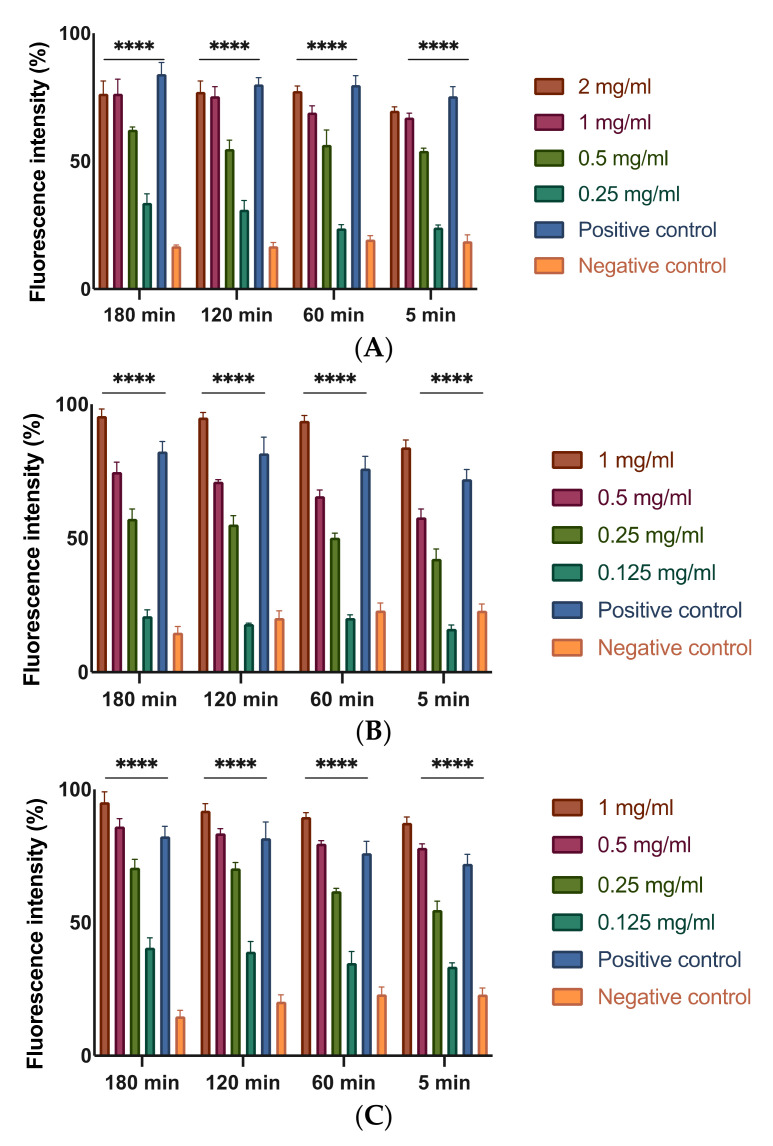
Effects on membrane permeability: (**A**) HA-MOE; (**B**) MeOH-MOE; (**C**) HAMD-MOE. The graph shows the loss of membrane integrity by the increase in fluorescence intensity in the treated samples. Data are the mean of 3 independent experiments performed on triplicate (mean +/− standard deviation), and values are represented as a percentage; **** *p*-values < 0.001.

Based on the results, it was evaluated whether extracts having a proven antibacterial activity act by modifying the permeability of the bacterial membrane. To evaluate this effect, different concentrations of each extract were tested on bacterial suspensions at different time points. To demonstrate the possible alteration of the membrane, a fluorescent intercalant agent, propidium iodide (PI), was added to EA bacterial suspensions: PI is not permeable to a healthy membrane, but when the integrity and permeability of that structure changes, PI enters the bacterial cell and intercalates between DNA bases, becoming detectable. As expected, at the MIC concentration of MOEs, there is an alteration of the permeability that is equivalent and even higher (for MeOH-MOE and HAMD-MOE) compared to the positive control represented by the bacteria treated with bleach (Figure 1). It is notable that, also at lower concentrations, such as 1/2 of the MIC, there is an increase in membrane permeability relative to PI, although it less significant, which indicates that even low concentrations of the extracts undermine the integrity and the permeability of the bacterial membrane.

### 2.3. In Vitro Assessment of Bacterial Swarming

Among the virulence factors of EA the activity of the external appendages is extremely important, especially in the phase of invasion of the plant vascular system, such as pili and flagella. To verify if MOEs were capable of inhibiting bacterial swarming, petri dishes were prepared by mixing soft LB agar (0.4% agarized soil), nitro blue tetrazolium chloride as a dye and each MOE at concentrations lower than the MIC. Then, a bacterial suspension was inoculated into the center of the petri dish. The soft agar allowed the swarming of EA, and the movement was detected thanks to the activity of nitro blue tetrazolium chloride: This compound can interact with the enzyme NADPH-oxidase, an enzyme capable of transferring electrons alternately to O_2_, with H_2_O_2_ formation, or to salt, with formation of an insoluble blue–black precipitate (formazan).

**Figure 2:**
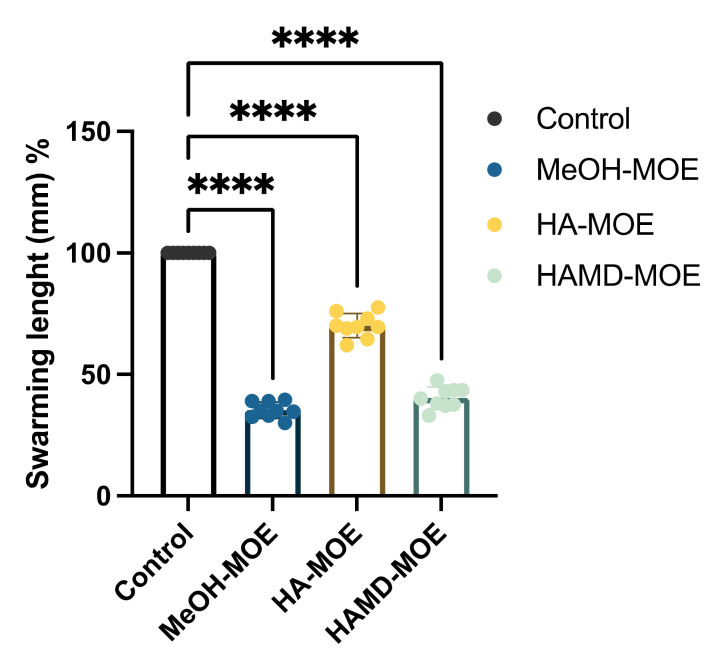
Effects of MOEs on swarming motility. EA movement is measured in mm; the measurement of the swarming area was taken from the point of inoculation and presented as the percentage compared to the control. Data are the mean of 3 independent experiments performed on triplicate (mean +/− standard deviation), and values are represented as a percentage; **** *p*-values < 0.001.

**Figure 3:**
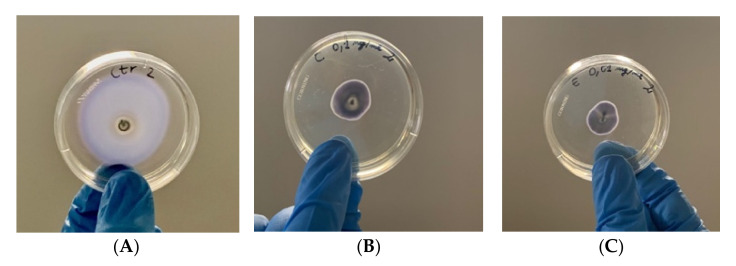
Effects of MOEs on swarming motility; images represent soft-agar plates inoculated with EA; the colored area is formed by formazan metabolized by the swarmed bacteria. (**A**) Control; (**B**) MeOH-MOE 0.1 mg/mL; (**C**) HAMD-MOE 0.01 mg/mL.

As observed in the graph (Figure 2) and represented as an example in the pictures (Figure 3), HAMD-MOE, MeOH-MOE and HA-MOE caused an inhibition of the motility of 60%, 65% and 30% after 6 days compared to the control: In fact, both the length and the area of movement decreased significantly when the bacterium was in contact with the extracts. According to previous studies, this effect could be due to an ATP depletion in the bacterial cells: The interference of these molecules with the cytoplasmic membrane can stop the driving flow-force generated by protons and electrons, resulting in a dissipation of the membrane potential and an alteration of the electrochemical gradient, which are essential elements for the synthesis of ATP needed for movement and chemotaxis [26].

### 2.4. In Vitro Assessment of Biofilm Formation

**Figure 4:**
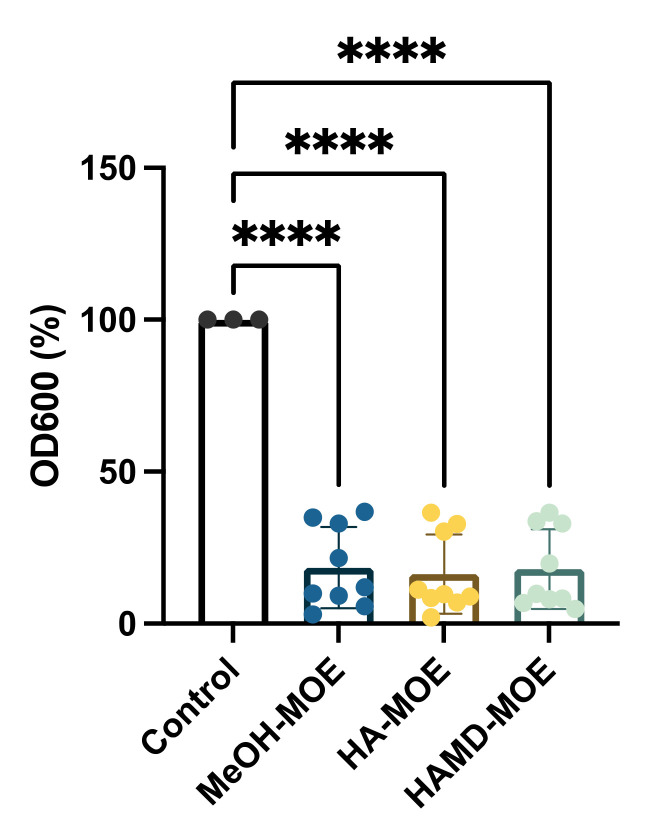
Effects of MOEs on biofilm formation: The EA biofilm was measured by OD_600_ and presented as the percentage compared to the control. Data are the mean of 3 independent experiments performed on triplicate (mean +/− standard deviation), and values are represented as a percentage; **** *p*-values < 0.001.

Biofilm formation is a phenomenon that allows bacterial colonies to exercise resistance in a more homogeneous and cohesive manner, thanks to the various intercellular communication mechanisms enhanced under stressful environmental conditions, and biofilm formation is an important step in fire blight pathogenesis. To assess whether the extracts exerted anti-biofilm activity, concentrations below the MICs of different MOEs were added to the bacterial suspensions. After incubation, biofilm formation was quantified by reading the plate with a spectrophotometer. In Figure 4, if compared to the positive control consisting only of the bacterial suspension with all MOEs tested, a significant decrease is notable in the formation of the biofilm. In this case, all extracts seem to act in the same way, reducing biofilm formation by 80%. The main accredited assumption is that certain phenolics (i.e., chlorogenic acid, quercetin, rutin, ellagic acid and others) may act as anti-biofilm compounds [24,27].

### 2.5. In Vitro Assessment of Amylovoran Production

**Figure 5:**
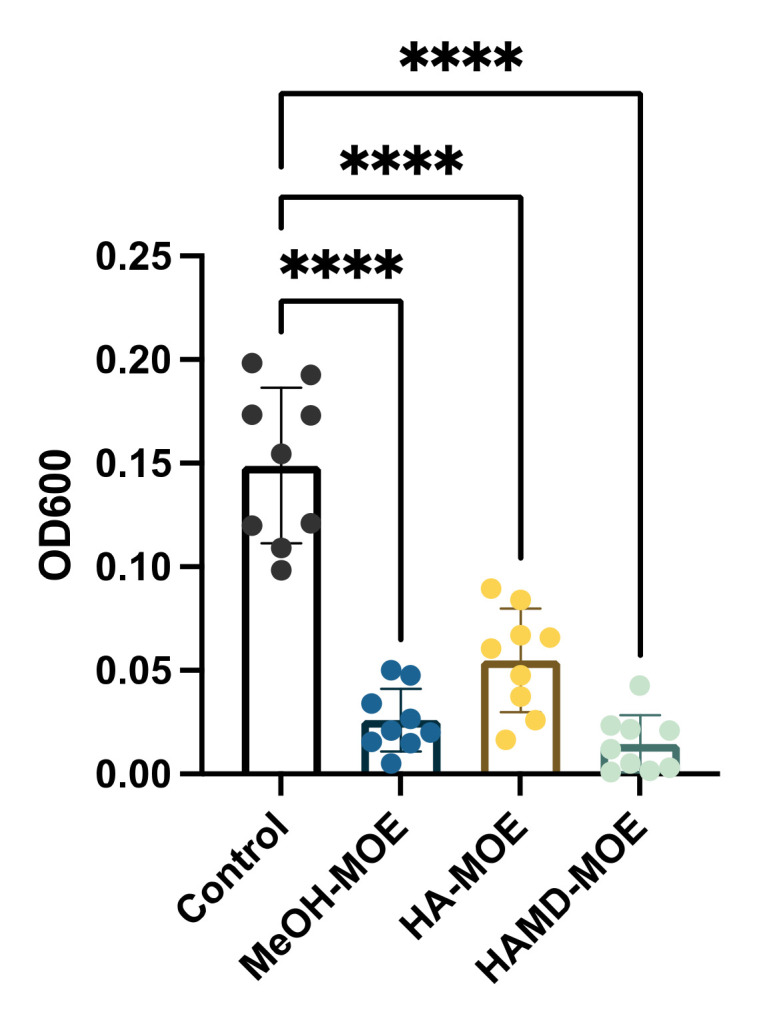
Effects of MOEs on amylovoran production. Amylovoran was measured by OD_600_. Data are the mean of 3 measurements performed on three different shoots (mean +/− standard deviation), and values are represented as a percentage; **** *p*-values < 0.001.

Aiming to obtain further insight into how virulence is impaired after exposure to the three MOEs, amylovoran production has been evaluated. MeOH-MOA, HA-MOE and HAMD-MOE resulted in a decrease in amylovoran synthesis of 84%, 63% and 93%, respectively. (Figure 5). EA virulence is dependent on to the production of amylovoran, and these findings demonstrate that the phytocomplex may target this virulence factor in order to establish all the effects demonstrated before [28,29]. No data are found about the ability of flavonoids or phenols to inhibit amylovoran production, and further studies are ongoing.

### 2.6. Antimicrobial Effects on Apple Trees

To define the capacity of HA-MOE, HAMD-MOE and MeOH-MOE to act against EA, a preliminary study was conducted to investigate the antibacterial effect on a plant system. Such an experiment can give us indications on how the bacterium interacts with plant cells and on the effect of MOEs in a three-dimensional, realistic context. The infected plant, in fact, reacts to the attack of the microorganisms activating defensive mechanisms that are innate or acquired, which come into play at different levels [28,30].

**Figure 6:**
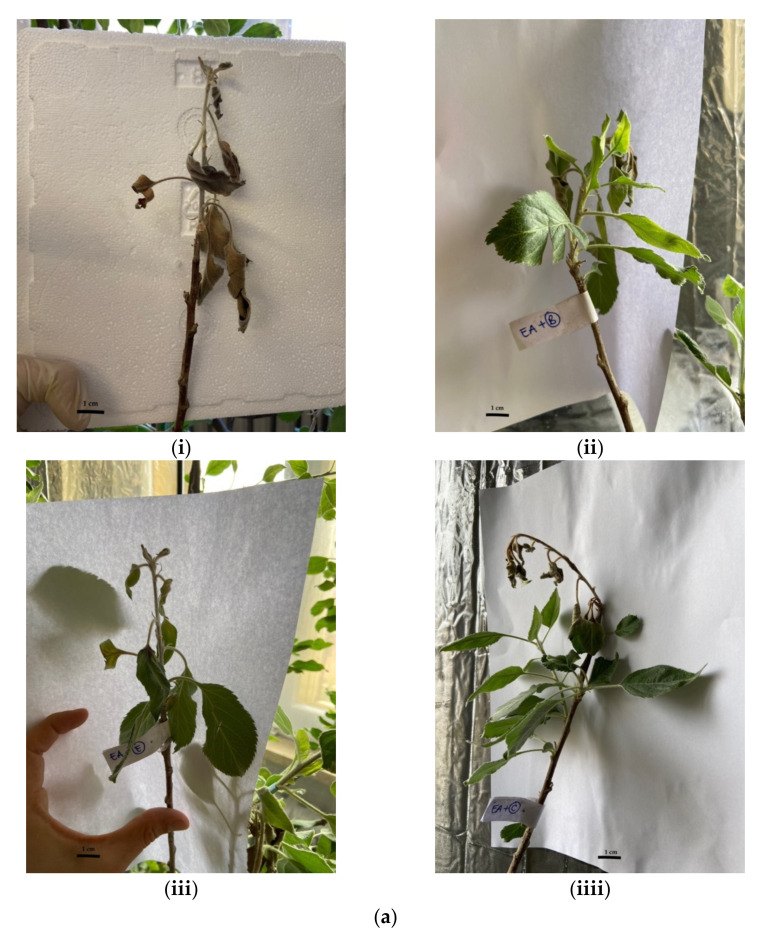

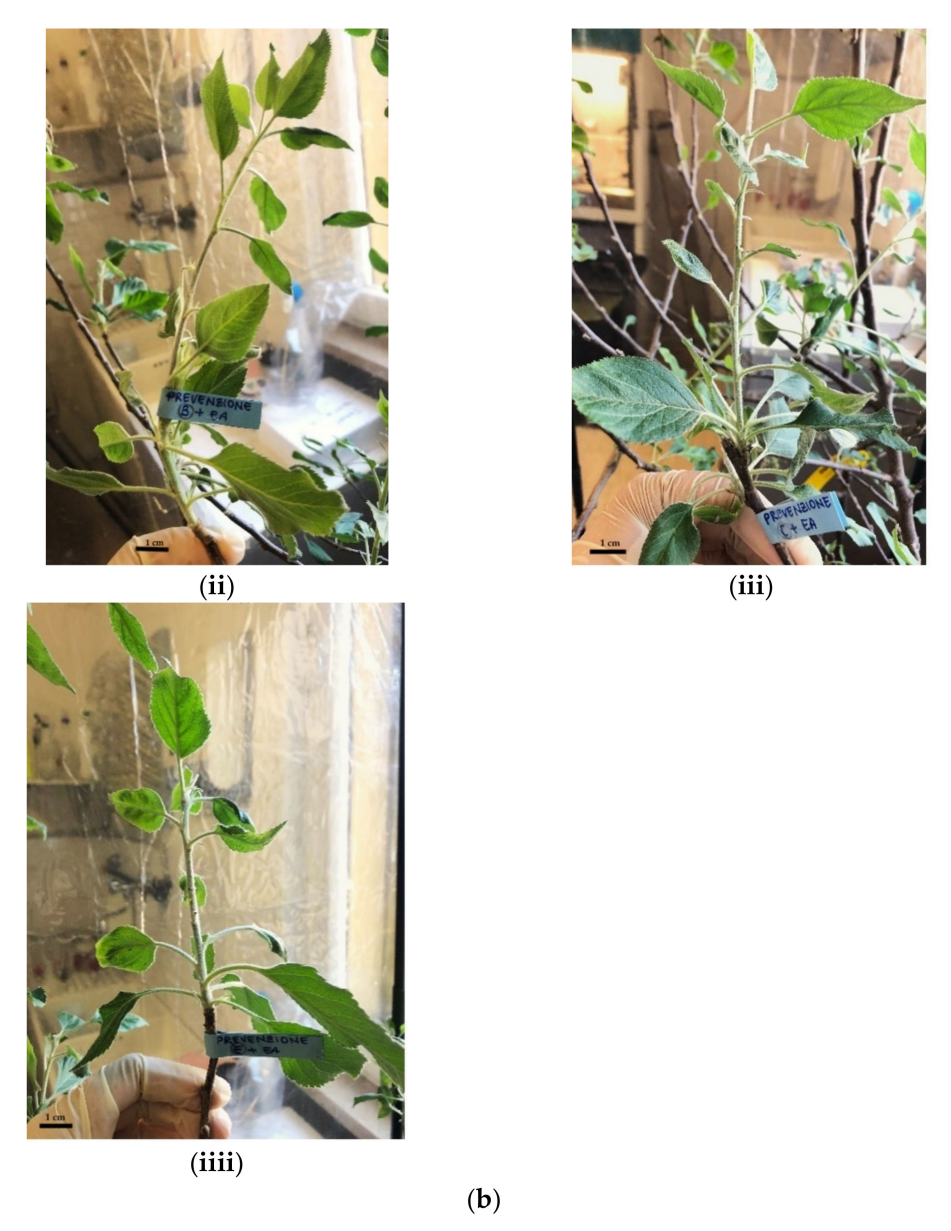
(**a**) **Therapeutic approach:** Effects of MOEs in infected apple shoots and treated with nebulized extracts after 48 h and 7 days post infection: (**i**) control. Therapeutic TREATMENTS: (**ii**) HA-MOE, (**iii**) HAMD-MOE, (**iiii**) MeOH-MOE. (**b**) **Preventive approach:** Effects of MOEs in healthy apple shoots nebulized with the extracts and then infected with EA after 24 h post treatments. (**i**) in (**a**) Control. Preventive TREATMENTS: (**ii**) HA-MOE; (**iii**) HAMD-MOE; (**iiii**) MeOH-MOE.

**Figure 7:**
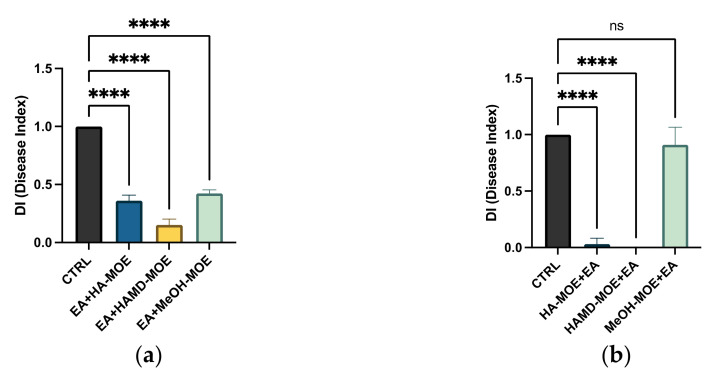
Effects of MOEs in infected apple shoots: (**a**) treatment after infection (therapeutic) and (**b**) treatment before infection (preventive). The EA infection in apple trees was measured by ImageJ quantification of the symptomatic area in the shoots and presented as the percentage compared to the control. Data are the mean of 3 measurements performed on three different shoots (mean +/− standard deviation), and values are represented as a percentage; **** *p*-values < 0.001.

As EA is the causative agent of fire blight in apple and pear trees, we tested the antimicrobial properties of such extracts on apple shootings previously infected with the bacterium. At the end of the treatment period, branches were cut to quantify the bacterial population within xylem and phloem. As observed in Figure 6a,b and Figure 7a,b, although all the tested extracts reduced the infection with significance, only the prevention treatment was able to eradicate it almost completely, as some wilting symptoms were still observed after the therapeutic treatment. Anyhow, the maltodextrin extract seems to be the most effective, reducing the wilting area by 80% compared to the control; also, MeOH-MOE and HA-MOE had shown a reduced infected area by 65% and 71%, respectively (Figure 7a,b). These results can give us important indications on which are the possible candidate molecules that exert this antibacterial effect; however, in view of a possible application in the agricultural field, this affirmation requires further analysis.

## 3. Discussion

The analysis of the minimum inhibitory concentration was carried out, showing a bacteriostatic effect of the different MOEs on all the tested strains. The results obtained show that the infusion and aqueous extracts with maltodextrins do not show antibacterial activity at any of the concentrations tested, while the hydroalcoholic extract, the hydroalcoholic with maltodextrin and methanolic extracts showed bacteriostatic and bactericidal effect at a concentration of 1 mg/mL. The enhanced effect is due to the extraction method: By using a solvent of an alcoholic nature, for the principle of molecular affinity, it allows the obtainment of extracts that are much more concentrated in terms of bioactive molecules. As was shown in our previous study, the most present active ingredients were found to be ferulic acid, rutin and chlorogenic acid. HA-MOE, HAMD-MOE and MeOH-MOE are comparable in terms of activity and in terms of polyphenol percentage, but the highest effect observed when using the maltodextrin hydroalcoholic extract is probably due to the presence of maltodextrins. These polysaccharides added as processing aid in spray drying processes, as they act as coating agents protecting bioactive molecules, prolonging their shelf life and preventing their loss of activity, particularly chlorogenic acid [31,32].

The activity is nevertheless being carried out by the high quantities of bioactive molecules found in the phytocomplex.

Based on data extracted from the literature and from our previous article, the antimicrobial effect can be attributed to the presence of many polyphenolic compounds, which act in a potentially synergistic manner [24]. It has been hypothesized that some of these compounds are able to complex at bacterial cell walls, altering fluidity, structure and functionality of the phospholipid double layer, resulting in a stop of ATP synthesis and a consequent slowdown of all ATP-dependent functions; simultaneously, the entrance through the altered membrane of compounds capable of inhibiting the enzyme complexes involved in bacterium replication is promoted [33,34,35]. Specifically, among the various phenols, flavonoids and phenolic acids detected in MOEs, rutin, naringenin, chlorogenic acid, ferulic acid and ellagic acid have been identified as compounds capable of complexing at bacterial cell walls, while robinetin, myricetin and epigallocatechin, once passing the bacterial membrane, seem to act by forming hydrogen bonds with nucleic bases, inhibiting the synthesis of DNA and RNA [34]. Flavonoids, such as quercetin and kaempferol, act by inhibiting DNA synthesis, blocking the ATP binding site of DNA gyrase, while others, such as morine, myricetin and luteolin, appear to inhibit the activity of the helicase, compromising cell division and the completion of chromosomal replication and resulting in inhibition of bacterial growth. Myricetin also appears to inhibit several key enzymes such as dihydrofolate reductase and several DNA- and RNA-polymerases. Finally, the glycosylate flavonol rutin can inhibit topoisomerase enzyme type II [27,36].

For this reason, an experiment has been carried out to assess the alteration of the integrity and permeability of the bacterial membrane of EA strains by MOEs, finding a great increase in permeability and the inhibition of cell growth by all three alcoholic extracts of *M. oleifera* at different times and concentrations, confirming the hypothesis described above. Although the mechanism of action by which such molecules act is not yet fully understood, some studies hypothesize a structure-related activity by which molecules with highly lipophilic portions interact with the lipids of the phospholipid layer and molecules with high redox potential interact with membrane proteins, resulting in a distortion of lipid–protein interaction [27,34,37,38]. In particular, phenolic compounds rich in alkyl chains of lipophilic nature seem to interact with membrane lipids by aligning with the fatty acid chains, thus dissolving in the phospholipid double layer. This distortion of the physical structure would facilitate the formation of channels, causing an increase in the degree of permeability [39,40]. Instead, molecules such as hydroxylated phenols, flavonols and quinones, characterized by both lipophilic portions and hydroxyl groups linked to an aromatic nucleus and, therefore, have a high redox potential, seem to irreversibly complex with membrane proteins, reacting with the sulfhydryl groups of their amino acids or through non-specific interactions, causing denaturation [33].

Hydrolysable tannins, such as ellagic acid, appear to complex non-specifically to membrane proteins through hydrogen bonds, covalent bonds and hydrophobic interactions, causing the deactivation of adhesins and membrane transport systems [41]. In addition, phenolic molecules such as rutin, quercetin, kaempferol and catechins appear to act, also inhibiting various enzymes involved in the biosynthesis of structural elements, especially fatty acids and peptidoglycan such as the enzyme FAS II (Fatty Acid Syntase II), the FAS II enzyme regulating protein transaclases, 3-ketoacyl-ACP-reductase and ACP-enoil-reductase; this results in blocking the synthesis of membrane phospholipids and the repair of any damage to the membrane [27]. The alteration of the integrity and permeability of the membrane leads to a considerable dissipation of energy, and this has, therefore, consequences on various ATP-dependent mechanisms, such as motility. From the results of the tests carried out on motility, by subjecting EA to MOEs, it found itself in conditions of energy scarcity such that the displacement length was significantly shorter than that of the untreated bacterium.

The lower efficiency of all ATP-dependent mechanisms also implies the deceleration, if not cessation, of all processes involved in the secretion of molecules necessary for intercellular communication and, therefore, amylovoran synthesis and biofilm formation. This hypothesis, probably, would explain the results obtained in the respective tests, which show a significant reduction in both amylovoran synthesis and biofilm formation. Regarding the anti-biofilm effect of extracts, it has been shown that different polyphenols such as phenolic acids, flavonoids, hydrolysable tannins and catechins inhibit the formation of biofilm by influencing the ATP-dependent mechanisms of bacterial regulation, such as quorum-sensing or other global regulatory systems. In other studies, the ability of some catechins and some flavonols to effectively counteract cellular adhesion, the initial stage of biofilm formation, in *E. coli* and *S. aureus* was found. Ellagic acid, tannic acid and epigallocatechin gallate, on the other hand, seem to inhibit the maturation phase of the biofilm, an effect probably related to the damage of the wall, mainly due to the cleavage of the peptidoglycan [34,35,36,39,42,43,44].

The impact of phenols and flavonoids on amylovoran production has not been tested yet, and further studies are needed to confirm the involvement of this virulence factor both in vitro and in planta. As the in planta experiments show, EA virulence is attenuated when exposed to these compounds; this effect is due to the suppression of T3SS: in 2013, Khokhani et al. identified phenolic compounds (benzoic acid and 4-methoxy-cinnamic acid) that were able to specifically target T3SS [29].

### Focus on Virulence Factors and Amylovoran Production

Our study lately focused on amylovoran production. Amylovoran production plays a huge role in the pathogenesis of fire blight and is one of the main virulence factors of EA. Amylovoran, a heteropolymer composed by monomeric units of glucuronic acid and galactose, has in fact being associated with biofilm formation processes. Different studies elucidate how amylovoran production is strictly connected to pathogen virulence: Yuan et al. discovered that plasmid pEA29 (a nearly ubiquitous plasmid) is required for EA to produce amylovoran and to form a biofilm, as mutants lacking the plasmid (amylovoran-deficient mutants) were completely non-pathogenic [5].

The effect of polyphenols on amylovoran production has not been investigated yet, but our results suggest that this is one of the main pathways involved in MOEs’ activity: In this study, we demonstrate that MOEs negatively affect EA virulence in vitro and in planta, as it was confirmed that amylovoran is needed for pathogenicity on immature pear fruits and pear plants [45,46].

**Figure 8:**
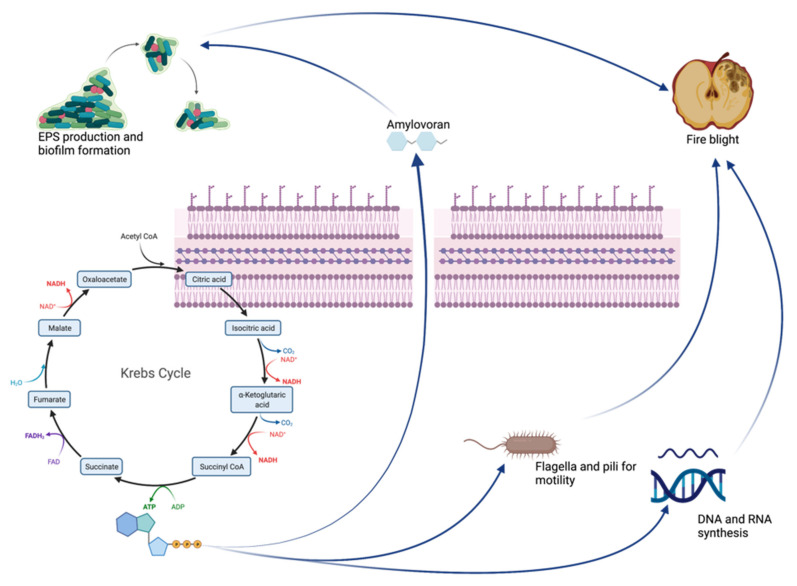
Fire blight pathogenesis and its main virulence factors: As previously reported, fire blight pathogenesis is strictly correlated to biofilm formation, swarming motility and EPS production. The ATP produced in the Krebs Cycle is fundamental for providing energy for all active processes within bacterial cells. Amylovoran permeabilization through the membrane results in EPS formation and biofilm production.

**Figure 9:**
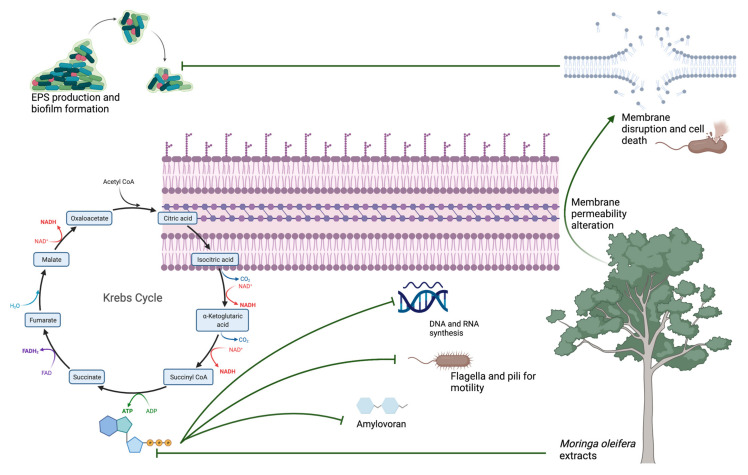
MOEs effects on EA virulence factors: as previously described, ATP shortening results in a halt in DNA and RNA synthesis, an inhibition of amylovoran production and a stop in motility. As the amylovoran is no longer produced, biofilm formation and consequent bacterial dissemination are impaired. Phenol’s effect of augmenting membrane permeability finally results in membrane disruption. All the summarized effects finally result in an inhibition of EA pathogenesis.

Amylovoran has also been connected to the YqhC transcriptional regulator, which is necessary for full virulence as it controls swarming motility and the production of amylovoran and siderophores, which were impaired by our MOEs [47]. Amylovoran has been well characterized in EA because of its essential role in disease development [46]. To cause disease on plants, EA cells move through plant vessels; bacterial aggregation and accumulation of amylovoran lead to the disruption of the water flow in the xylem. The leakage of the vessels and extrusion of the bacteria into the parenchyma causes bacteria to leak onto the surface of the plants and ultimately results in the wilting of the shoots [48]. Therefore, bacterial motility and amylovoran production are closely related to pathogenicity [4]. In the current study, the MOE-treated strain showed a significant reduction in motility, biofilm formation, altered membrane permeability and a reduction in amylovoran production compared with the control. Lesion length produced on apple shoots treated with the MOEs was also significantly reduced. It must be remembered that amylovoran is produced during all stages of bacterial growth and does not stop in the stationary phase, where the accumulation of amylovoran is the greatest. Once secreted by EA, it localizes around the bacterial membrane itself, encapsulating it. From here, it is then gradually released as free amylovoran, especially under stress conditions: In vitro studies have shown that the concentration of amylovoran was higher when samples were subjected to mechanical stress and low osmotic pressure or low temperatures [49,50]. From the results of Ricardo D. Santander and Elena G. Biosca of 2017, it was in fact discovered that amylovoran was produced more intensely around 4 °C, while levan production and the formation of a biofilm had a peak at 14 °C [50]. At the same time, a decrease in the levels of amylovoran and levan in time was found, which could prove that these EPSs are used as an alternative source of carbon nourishment [51]. The discrepancy between the different levels of virulence observed at different temperatures, however, makes us understand the complexity of the interaction between EA and its host. Subsequently, it has been shown that temperature determines two types of responses under stress conditions induced by a nutrient-poor environment (oligotrophic soil): At temperatures below 14 °C, a state called “starvation-survival” is mainly induced, in which the cells remain “culturable” and virulent, while at temperatures higher than 28 °C, a state called “viable but nonculturable” (VBNC response) is introduced, in which bacterial cells remain viable but do not grow and do not create new colonies [50,51]. Our experiments confirm these different states of EA, as amylovoran production has been highly induced when bacteria have been cultured at 4 °C, but they are still limited by the effects of MOEs. It is, therefore, assumed that starvation-survival and VBNC states are physiological states of EA and are part of its life cycle. In summary, the purpose of the increased synthesis of amylovoran as an adaptive response to various types of stress can be to act as a nutrient source of carbon, elude the immune response of the host plant masking the bacterium and, by contributing to the formation of biofilms, to enhance virulence and protect bacteria from drying, toxic compounds (such as chlorine or copper or from bacteriophages) and to favor the starvation-survival state. Fire blight infection and MOEs effects are briefly summarized in Figure 8 and Figure 9.

Indeed, we can state that the inhibition of the synthesis of amylovoran and the effect of phenols on permeability result in all the effects studied in our project and to a halt of the fire blight disease. The in planta experiments show us the ability of MOEs to stop the spread of the infection, both if used as a preventive and as a therapeutic measure, allowing us to state that the bacteria do not actively colonize the xylem. This effect might be due to amylovoran inhibition and to biofilm formation inhibition. The involvement of the T3SS system, which is only active when the bacterium encounters its host, still needs to be evaluated.

## 4. Materials and Methods

### 4.1. Erwinia amylovora Strains and Culture Conditions

EA isolates used in this study were donated from the Emilia-Romagna Phytosanitary Agency, and strain 30165 from Leibniz Institute DSMZ-German Collection of Microorganisms was used as a control. The strains were isolated from pear and apple trees from six provinces of the Region, as specified in Table 2. Stocks of the bacterial strains were conserved at −80 °C in Luria-Bertani (LB) broth with 50% glycerol. During the study, bacteria were plated on LB agar (Liofilchem, Roseto degli Abruzzi, TE, Italy, 30 g/L) and incubated at 25 °C/28 °C. The different concentrations of the inoculum used in the experiments derive from the literature’s data and protocols cited in the respective paragraphs. In the case of biofilm formation, a bigger inoculum is usually used to obtain faster results and to ensure the biofilm formation processes itself. The same reasoning lays behind the choice to use a high concentrated inoculum for in planta experiments. For the membrane permeability and the swarming motility assay, the concentrations of PI and TCC used were usually tested on a bacterial concentration ranging from 10^4^ to 10^7^; thus, we tried different concentrations, choosing an inoculum of 10^5^ for the standardized experiments, in accordance with the literature and based on the performance of our experiments.

### 4.2. Determination of the Minimum Inhibitory Concentration (MIC) of Different M. oleifera Lam. Extracts

MIC, defined as the lowest concentration of a chemical molecule that blocks visible bacterial growth after overnight incubation, was determined. To determine MIC, the microdilution method was used, as described by Akhlaghi et al. [45]. EA was cultured in LB broth overnight at 28 °C, 160 rpm in a shaking incubator (MaxQ 4000, Thermo Scientific Italia, Milano, Italy). MOEs measuring 80 µL (5 mg/mL stock concentration) were added to 120 µL of LB broth in order to obtain a 2 mg/mL concentration of MOEs in the first well of each row in 96-well plates (Costar Corning, Corning, NY, USA). The extracts were then diluted in the 96-well microplate to obtain a range of concentrations from 2 mg/mL to 0.001 mg/mL, in a total volume of 200 µL. Then, 10 µL of the overnight culture was inoculated into each well and standardized with a 10^4^ CFU/mL inoculum. The microplate was then statically incubated for 48 h at 25 °C. Extracts were all dissolved in methanol and diluted with water down to a methanol concentration of 0.1% *v*/*v*. To evaluate the influence of this concentration of methanol, controls differed according to the dilutions of the extracts and, thus, of methanol. The MOEs’ concentrations we then used for the other experiments had a methanol concentration of 0.001%, and its effects on bacterial growth were not detectable and non-significant. All the analysis were performed from data obtained from three different experiments in triplicate.

### 4.3. Membrane Permeability Assay

EA bacterial suspensions were grown overnight in LB broth for 24 h at 25 °C. After incubation, 1 × 10^5^ CFU/mL of bacteria was placed in four different eppendorfs containing *M. oleifera* Lam. extracts at concentrations corresponding to 1 × MIC, 1/2 × MIC and 1/4 × MIC. These suspensions were incubated for 180 min, 120 min, 60 min and 5 min. After the incubation time, the suspensions were centrifuged for 5 min at 14,000 rpm and then washed with PBS 1×. The pellet was then resuspended with propidium iodide (0.5%) and incubated for 15 min, avoiding exposure of the suspension to light sources. Then, each suspension has been plated into 96-well plates, and the values were read through a fluorescence microplate reader (Tecan-Fluoroscan, Tecan Italia, Cernusco sul Naviglio, MI, Italy) [52]. Negative control consisted in untreated EA cells, while positive control consisted in EA treated with 10% bleach solution. All analyses were performed from data obtained from three different experiments in triplicate.

### 4.4. Swarming Motility Assay

Bacterial suspensions were grown overnight in LB broth for 24 h at 28 °C. After incubation, bacteria were centrifuged and washed three times with PBS 1× (Sigma Aldrich, St. Louis, MO, USA). Then, the pellet was resuspended in PBS 1× and bacterial suspensions were diluted in water (1:10 dilution), and 10 µL (corresponding to 1 × 10^5^ CFU/mL) of the diluted suspension was plated in the center of a LB soft-agar plate (agar 0.4%) containing MOEs in non-lethal concentrations, as described by Chen et al. [53] The swarming area was then quantified and compared to the untreated control by measuring the diameter of the swarmed zone and measuring the length from the inoculation point to the edge of the swarmed zone, after 48 and 72 h of incubation at 25 °C using Fiji software [54]. All analyses were performed from data obtained from three different experiments in triplicate.

### 4.5. Biofilm Formation

EA forms biofilms that represent a critical biological process in the pathogenic cycle of fire blight disease. The effects on biofilm formation were determined by the microplate assay with crystal violet, as described by Wilson et al. [55]. EA suspensions, containing 10^6^ CFU/mL, were inoculated in LB broth with MOEs at non-lethal concentration in a 96-well U-bottom microplate for 72 h at 25 °C. After the incubation time, the growth media, MOEs and planktonic cells were removed from the plate and washed with sterile deionized water. Crystal violet 1% was added to each well and incubated for 30 min at room temperature. Then, the dye solution was removed by washing the plate several times with deionized water. Decoloring solutions measuring 200 µL (90–95% ethanol) were then added to each well and incubated for 15 min at room temperature to increase crystal violet solubility. The 96-well plate content was then transferred to a new clean microplate, and biofilm formation was quantified and compared to the untreated control by reading the absorbance at 570 nm in a microplate reader (Tecan-Sunrise, Tecan Italia, Cernusco sul Naviglio, MI, Italy). All analyses were performed from data obtained from three different experiments in triplicate.

### 4.6. Amylovoran Production

Amylovoran is one of the major virulence factors of EA: In fact, pathogenesis is dependent on the expression of the T3SS and the production of the exopolysaccharide (EPS) amylovoran. To assess amylovoran production, the cetylpyrimidinium chloride (CPC) assay had been used, as described by Bellemann et al. [49]. EA cold overnights pellets were washed with PBS 1X and inoculated 1:100 in MBMA medium (3 g of KH_2_PO_4_, 7 g of K_2_HPO_4_, 1 g of [NH_4_]_2_SO_4_, 2 mL of glycerol, 0.5 g of citric acid and 0.03 g of MgSO_4_) containing 1% sorbitol. MBMAs supernatants were tested for amylovoran concentration by incubating with 50 µL of 50 mg/mL CPC per milliliter of supernatant for 10 min. The control consisted in untreated EA cells. Turbidity was then measured with a spectrophotometer at OD_600_. All analyses were performed from data obtained from three different experiments in triplicate.

### 4.7. Effects of MOE on Apple Trees

EA strains were grown overnight in 5 mL of LB broth at 28 °C for 24 h, centrifuged and then resuspended with 1 × PBS to reach a bacterial concentration of 1 × 10^7^ CFU/mL. To assay the pathogenicity of apple seedlings, two-year-old apple plants (*Malus domestica* ‘Gala’) were used for greenhouse experiments. During the experiments, plants were grown at a continuous temperature of 25 °C and 70% relative humidity, under 12 h of sunlight every day. For the control, 50 μL (1 × 10^7^ CFU/mL) of EA suspension was inserted into the shoots (15–20 cm) by syringe inoculation. For the preventive treatment protocol, MOEs at their MIC concentration were sprayed on every shoot, every 48 h, for one week; then, 50 μL of EA suspension was inserted into the shoots (15–20 cm) by syringe inoculation. For the treatment protocol, 50 μL of EA suspension was first inoculated on the sprouts (15–20 cm) by using a syringe, and 48 h later, the different MOEs were sprayed on each infected sprout (except the control). Following 14 and 28 days after the inoculation of EA, disease symptoms were recorded. The disease symptoms were developed on the shoots. Total shoot lengths and lesion lengths were determined for each shoot. Fire blight shoot susceptibility was computed using the disease index (DI) as follows: DI = (Length of blighted shoot/Total shoot length) × 100 [30]. The experiment was carried out in three replicates.

### 4.8. Plant Material and Extraction Methods

*M. oleifera* Lam. leaves were harvested in August 2019 (Lot number: 19E0854X1809). After collection, leaves were dried by Evra S.r.l. (Loc. Galdo, 85044 Lauria, Italy). The dried sample was packaged and sent to our laboratory. Upon arrival, dried leaves were ground to a fine powder with a mortar and stored at −80 °C. Extracts were stored at −18 °C until use. The water and the hydroalcoholic extracts with maltodextrin (WMD-MOE and HAMD-MOE, respectively) were obtained by Evra S.r.l. company. Extraction methods were performed as described in Fontana et al. [24]:

- Hydroalcoholic extract (HA-MOE): Leaf powder was mixed with hydroalcoholic solution (ethanol:water, 70:30) at room temperature, filtered and concentrated in vacuum.
- Methanolic extract (MeOH-MOE): Leaf powder was mixed with methanol and subjected to two sonication cycles (40 °C, 60 min, 80%) with subsequent centrifugation and concentrated under vacuum.
- Decoction (In-MOE): Leaf powder was left to infuse for 30 min with 150 mL of de- ionized water, previously brought to a boil. The decoction solution was filtered and lyophilized.
- Water extract with maltodextrins (WMD-MOE): Dried leaves were extracted with water (raw material:solvent 1:10) for 45 min at 65 °C. After filtration, concentration and pasteurization, the extract was spray dried using maltodextrin, obtaining a fine powder.
- Hydroalcoholic extract with maltodextrins (HAMD-MOE): Dried leaves were extracted with 50% ethanol (raw material:solvent 1:10) for 45 min at 45 °C. After filtration, concentration and pasteurization, the extract was spray dried using maltodextrin, obtaining a fine powder.

### 4.9. Statistical Analysis

All tests were performed three times in triplicate, and statistical analysis was performed using one-way ANOVA followed by Dunnett’s multiple comparisons test with GraphPad Prism version 9.0.0 for MacOS (GraphPad Software, San Diego, CA, USA), with *p* ≤ 0.05 to identify significant differences.

## 5. Conclusions

Our results indicate that amylovoran production is significantly impaired when EA comes in contact with the different tested MOEs, resulting in a halt in EPS production and biofilm formation. All the results obtained in our experiments by MOEs on EA leads us to the hypothesis that the phenolic compounds in the phytocomplex firstly alter membrane permeability, allowing different molecules to permeate inside the cells; this results in a halt of all ATP-involving processes, inhibiting amylovoran production and, thus, swarming motility and biofilm formation.

In spring, young shoots infection is the main source of dissemination of EA, and the pathogen must establish a large epiphytic population on stigmas in order to successfully infect host plants. In fact, the control of fire blight is mainly obtained by applying antibiotics to flowers to suppress pathogen infection. By spraying apple shoots, we have shown that the phytocomplex can reduce disease development. These results suggest that amylovoran production inhibitors could be useful in the control of fire blight disease.

The aim to reduce the use of toxic products to avoid adverse effects on human health and on the environment, as well as the appearance of resistant bacteria, has prompted research to investigate the use of phytocomplexes with antimicrobial properties as an effective and eco-sustainable strategy. From our studies, therefore, the antimicrobial activity of the extracts of leaves of *M. oleifera* Lam. on EA looks promising: the multiple and synergic mechanisms of action involved with the phytocomplex; the high efficacy; eco-compatibility and sustainability that it would imply; low cost and low risks; and the in planta efficacy make these extracts a platform for controlling fire blight.

## Figures and Tables

**Table 1:** MIC values: Each value was obtained from three different experiments performed on triplicate (mean +/− standard deviation). No significant difference was observed for each EA strain; reason why the results are presented with one value for all strains.

*M. oleifera* Lam. Leaves Extract	MIC (mg/mL)
Infusion (In-MOE)	>2
Hydroalcoholic extract (HA-MOE)	1
Methanolic extract (MeOH-MOE)	1
Water extract with maltodextrins (WMD-MOE)	>2
Hydroalcoholic extract with maltodextrins (HAMD-MOE)	1

**Table 2:** List of EA bacterial isolates.

BACTERIA	Sample ID	Host	Province of Isolation	Year of Isolation
*Erwinia amylovora*	49536	Apple tree	Forlì-Cesena	2018
*Erwinia amylovora*	49540	Apple tree	Reggio Emilia	2018
*Erwinia amylovora*	50163	Apple tree	Ravenna	2018
*Erwinia amylovora*	50340	Apple tree	Ravenna	2018
*Erwinia amylovora*	50535	Pear tree	Piacenza	2018
*Erwinia amylovora*	51198	Pear tree	Ravenna	2018
*Erwinia amylovora*	51201	Pear tree	Ravenna	2018
*Erwinia amylovora*	51431	Pear tree	Piacenza	2018
*Erwinia amylovora*	51657	Pear tree	Ravenna	2018
*Erwinia amylovora*	52140	Apple tree	Bologna	2018
*Erwinia amylovora*	52458	Pear tree	Ferrara	2018
*Erwinia amylovora*	30165	Pear tree	DSMZ	Before 22 August 1990

## Data Availability

Not applicable.

## References

[1] Kharadi R.R., Sundin G.W. (2021). Dissecting the process of xylem colonization through biofilm formation in *Erwinia amylovora*. J. Plant Pathol..

[2] Zeng Q., Puławska J., Schachterle J. (2021). Early events in fire blight infection and pathogenesis of *Erwinia amylovora*. J. Plant Pathol..

[3] Slack S.M., Zeng Q., Outwater C.A., Sundin G.W. (2017). Microbiological Examination of *Erwinia amylovora* Exopolysaccharide Ooze. Phytopathology.

[4] Piqué N., Miñana-Galbis D., Merino S., Tomás J.M. (2015). Virulence Factors of *Erwinia amylovora*: A Review. Int. J. Mol. Sci..

[5] Yuan X., McGhee G.C., Slack S.M., Sundin G.W. (2021). A Novel Signaling Pathway Connects Thiamine Biosynthesis, Bacterial Respiration, and Production of the Exopolysaccharide Amylovoran in *Erwinia amylovora*. Mol. Plant-Microbe Interact..

[6] Mendes R., Regalado L., Luz J., Tassi N., Teixeira C., Gomes P., Tavares F., Santos C. (2021). In Vitro Evaluation of Five Antimicrobial Peptides against the Plant Pathogen *Erwinia amylovora*. Biomolecules.

[7] Gusberti M., Klemm U., Meier M.S., Maurhofer M., Hunger-Glaser I. (2015). Fire Blight Control: The Struggle Goes On. A Comparison of Different Fire Blight Control Methods in Switzerland with Respect to Biosafety, Efficacy and Durability. Int. J. Environ. Res. Public Health.

[8] Ivanović M., Gašić K., Prokić A., Kuzmanović N., Zlatković N., Obradovic A. (2016). Screening for copper and antibiotic resistance in *Erwinia amylovora* population from Serbia. Acta Hortic..

[9] Smith D.D.N., Williams A.N., Verrett J.N., Bergbusch N.T., Manning V., Trippe K., Stavrinides J. (2019). Resistance to Two Vinylglycine Antibiotic Analogs Is Conferred by Inactivation of Two Separate Amino Acid Transporters in *Erwinia amylovora*. J. Bacteriol..

[10] Mikiciński A., Puławska J., Molzhigitova A., Sobiczewski P. (2019). Bacterial species recognized for the first time for its biocontrol activity against fire blight (*Erwinia amylovora*). Eur. J. Plant Pathol..

[11] Buttimer C., McAuliffe O., Ross R.P., Hill C., O’Mahony J., Coffey A. (2017). Bacteriophages and Bacterial Plant Diseases. Front. Microbiol..

[12] Nazzaro F., Fratianni F., De Martino L., Coppola R., De Feo V. (2013). Effect of Essential Oils on Pathogenic Bacteria. Pharmaceuticals.

[13] Abdalla A.M., Alwasilah H.Y., Al R., Mahjoub H., Mohammed I., Yagoub M. (2016). Evaluation of Antimicrobial Activity of *Moringa oleifera* Leaf Ex-Tracts against Pathogenic Bacteria Isolated from Urinary Tract Infected Patients. J. Adv. Lab. Res. Biol..

[14] Pal S.K., Mukherjee P.K., Saha K., Pal M., Saha B.P. (1995). Ancient Science of Life. Anc. Sci. Life.

[15] Ervianingsih, Mursyid M., Annisa R.N., Zahran I., Langkong J., Kamaruddin I. (2019). Antimicrobial activity of moringa leaf (*Moringa oleifera* L.) extract against the growth of Staphylococcus epidermidis. IOP Conference Series: Earth and Environmental Science.

[16] Rani N.Z.A., Husain K., Kumolosasi E. (2018). Moringa Genus: A Review of Phytochemistry and Pharmacology. Front. Pharmacol..

[17] Sreelatha S., Padma P.R. (2009). Antioxidant Activity and Total Phenolic Content of *Moringa oleifera* Leaves in Two Stages of Maturity. Mater. Veg..

[18] Atawodi S.E., Atawodi J.C., Idakwo G.A., Pfundstein B., Haubner R., Wurtele G., Bartsch H., Owen R.W. (2010). Evaluation of the Polyphenol Content and Antioxidant Properties of Methanol Extracts of the Leaves, Stem, and Root Barks of *Moringa oleifera* Lam. J. Med. Food.

[19] Dhakad A.K., Ikram M., Sharma S., Khan S., Pandey V.V., Singh A. (2019). Biological, nutritional, and therapeutic significance of *Moringa oleifera* Lam. Phytother. Res..

[20] Leone A., Fiorillo G., Criscuoli F., Ravasenghi S., Santagostini L., Fico G., Spadafranca A., Battezzati A., Schiraldi A., Pozzi F. (2015). Nutritional Characterization and Phenolic Profiling of *Moringa oleifera* Leaves Grown in Chad, Sahrawi Refugee Camps, and Haiti. Int. J. Mol. Sci..

[21] Sahakitpichan P., Mahidol C., Disadee W., Ruchirawat S., Kanchanapoom T. (2011). Unusual glycosides of pyrrole alkaloid and 4′-hydroxyphenylethanamide from leaves of *Moringa oleifera*. Phytochemistry.

[22] Chhikara N., Kaur A., Mann S., Garg M., Sofi S.A., Panghal A. (2020). Bioactive compounds, associated health benefits and safety considerations of *Moringa oleifera* L.: An updated review. Nutr. Food Sci..

[23] Elgamily H., Moussa A., Elboraey A., El-Sayed H., Al-Moghazy M., Abdalla A. (2016). Microbiological Assessment of *Moringa oleifera* Extracts and Its Incorporation in Novel Dental Remedies against Some Oral Pathogens. Open Access Maced. J. Med Sci..

[24] Fontana R., Caproni A., Buzzi R., Sicurella M., Buratto M., Salvatori F., Pappadà M., Manfredini S., Baldisserotto A., Marconi P. (2021). Effects of *Moringa oleifera* Leaf Extracts on *Xanthomonas campestris* pv. *campestris*. Microorganisms.

[25] Baldisserotto A., Buso P., Radice M., Dissette V., Lampronti I., Gambari R., Manfredini S., Vertuani S. (2018). *Moringa oleifera* Leaf Extracts as Multifunctional Ingredients for “Natural and Organic” Sunscreens and Photoprotective Preparations. Molecules.

[26] Zhao Y., Wang D., Nakka S., Sundin G.W., Korban S.S. (2009). Systems level analysis of two-component signal transduction systems in *Erwinia amylovora*: Role in virulence, regulation of amylovoran biosynthesis and swarming motility. BMC Genom..

[27] Mickymaray S. (2019). Efficacy and Mechanism of Traditional Medicinal Plants and Bioactive Compounds against Clinically Important Pathogens. Antibiotics.

[28] Yang F., Korban S.S., Pusey P.L., Elofsson M., Sundin G.W., Zhao Y. (2014). Small-molecule inhibitors suppress the expression of both typeIIIsecretion and amylovoran biosynthesis genes in *Erwinia amylovora*. Mol. Plant Pathol..

[29] Khokhani D., Zhang C., Li Y., Wang Q., Zeng Q., Yamazaki A., Hutchins W., Zhou S.-S., Chen X., Yang C.-H. (2013). Discovery of Plant Phenolic Compounds That Act as Type III Secretion System Inhibitors or Inducers of the Fire Blight Pathogen, *Erwinia amylovora*. Appl. Environ. Microbiol..

[30] Ozrenk K., Balta F., Çelik F. (2011). Levels of fire blight (Erwinia amylovora) susceptibility of native apple, pear and quince germplasm from Lake Van Basin, Turkey. Eur. J. Plant Pathol..

[31] Harsha P.S.S., Lavelli V. (2019). Effects of Maltodextrins on the Kinetics of Lycopene and Chlorogenic Acid Degradation in Dried Tomato. Molecules.

[32] Pettinato M., Aliakbarian B., Casazza A., Perego P. (2017). Encapsulation of antioxidants from spent coffee ground extracts by spray drying. Chem. Eng. Trans..

[33] Cowan M.M. (1999). Plant Products as Antimicrobial Agents. Clin. Microbiol. Rev..

[34] Cushnie T.P.T., Lamb A.J. (2005). Antimicrobial activity of flavonoids. Int. J. Antimicrob. Agents.

[35] Ikigai H., Nakae T., Hara Y., Shimamura T. (1993). Bactericidal catechins damage the lipid bilayer. Biochim. Biophys. Acta—Biomembr..

[36] Griep M.A., Blood S., Larson M., Koepsell S.A., Hinrichs S.H. (2007). Myricetin inhibits Escherichia coli DnaB helicase but not primase. Bioorgan. Med. Chem..

[37] Liu J., Du C., Beaman H.T., Monroe M.B.B. (2020). Characterization of Phenolic Acid Antimicrobial and Antioxidant Structure-Property Relationships. Pharmaceutics.

[38] Hossain S.I., Saha S.C., Deplazes E. (2021). Phenolic compounds alter the ion permeability of phospholipid bilayers via specific lipid interactions. Phys. Chem. Chem. Phys..

[39] Burt S. (2004). Essential oils: Their antibacterial properties and potential applications in foods—A review. Int. J. Food Microbiol..

[40] Marrufo T., Nazzaro F., Mancini E., Fratianni F., Coppola R., De Martino L., Agostinho A.B., De Feo V. (2013). Chemical Composition and Biological Activity of the Essential Oil from Leaves of Moringa oleifera Lam. Cultivated in Mozambique. Molecules.

[41] Scalbert A. (1991). Antimicrobial properties of tannins. Phytochemistry.

[42] Cushnie T.T., Cushnie B., Lamb A. (2014). Alkaloids: An overview of their antibacterial, antibiotic-enhancing and antivirulence activities. Int. J. Antimicrob. Agents.

[43] Muñoz-Cazares N., García-Contreras R., Pérez-López M., Castillo-Juárez I., Soto-Hernandez M., Palma-Tenango M., del Rosario Garcia-Mateos M. (2017). Phenolic compounds with anti-virulence properties. Phenolic Compounds—Biological Activity.

[44] Tsuchiya H., Sato M., Miyazaki T., Fujiwara S., Tanigaki S., Ohyama M., Tanaka T., Iinuma M. (1996). Comparative study on the antibacterial activity of phytochemical flavanones against methicil-lin-resistant *Staphylococcus aureus*. J. Ethnopharmacol..

[45] Akhlaghi M., Tarighi S., Taheri P. (2019). Effects of plant essential oils on growth and virulence factors of Erwinia amylovora. J. Plant Pathol..

[46] Koczan J.M., McGrath M.J., Zhao Y., Sundin G.W. (2009). Contribution of Erwinia amylovora Exopolysaccharides Amylovoran and Levan to Biofilm Formation: Implications in Pathogenicity. Phytopathol..

[47] Sahebi M., Tarighi S., Taheri P. (2021). The Arac-like transcriptional regulator YqhC is involved in pathogenicity of Erwinia amylovora. J. Appl. Microbiol..

[48] Borruso L., Salomone-Stagni M., Polsinelli I., Schmitt A., Benini S. (2017). Conservation of Erwinia amylovora pathogenicity-relevant genes among Erwinia genomes. Arch. Microbiol..

[49] Bellemann P., Bereswill S., Berger S., Geider K. (1994). Visualization of capsule formation by Erwinia amylovora and assays to determine amylovoran synthesis. Int. J. Biol. Macromol..

[50] Santander R.D., Biosca E.G. (2017). Erwinia amylovorapsychrotrophic adaptations: Evidence of pathogenic potential and survival at temperate and low environmental temperatures. PeerJ.

[51] Ordax M., Marco-Noales E., López M.M., Biosca E.G. (2006). Survival Strategy of Erwinia amylovora against Copper: Induction of the Viable-but-Nonculturable State. Appl. Environ. Microbiol..

[52] Crowley L.C., Scott A.P., Marfell B.J., Boughaba J.A., Chojnowski G., Waterhouse N.J. (2016). Measuring Cell Death by Propidium Iodide Uptake and Flow Cytometry. Cold Spring Harb. Protoc..

[53] Chen W., Mani S., Tang J.X. (2021). An Inexpensive Imaging Platform to Record and Quantitate Bacterial Swarming. Bio-Protocol.

[54] Schindelin J., Arganda-Carreras I., Frise E., Kaynig V., Longair M., Pietzsch T., Preibisch S., Rueden C., Saalfeld S., Schmid B. (2012). Fiji: An open-source platform for biological-image analysis. Nat. Methods.

[55] Wilson C., Lukowicz R., Merchant S., Valquier-Flynn H., Caballero J., Sandoval J., Okuom M., Huber C., Brooks T.D., Wilson E. (2017). Quantitative and Qualitative Assessment Methods for Biofilm Growth: A Mini-review. Res. Rev. J. Eng. Technol..

